# EHMTI-0332. Health care utilisation for primary headache disorders: insights from Karnataka, India

**DOI:** 10.1186/1129-2377-15-S1-D48

**Published:** 2014-09-18

**Authors:** G Rao, G Gururaj, G Kulkarni, DK Subbukrishna, T Steiner, L Stovner

**Affiliations:** 1 Epidemiology Centre for Public Health, National Institute of Mental Health and Neurosciences, Bangalore, India; 2 Neurology, National Institute of Mental Health and Neurosciences, Bangalore, India; 3 Bio-statistics, National Institute of Mental Health and Neurosciences, Bangalore, India; 4 Neuroscience, Imperial College, London, UK; 5 Neuroscience, Norwegian University of Science and Technology, Trondheim, Norway

## Background

Globally, primary headache disorders are neglected and inadequately treated. There is little information on management or health-care utilisation for headache disorders from the Indian sub-continent.

## Aim

To fill this knowledge gap in Karnataka State, south India.

## Methods

In a population-based survey, 2,329 randomly selected, biologically unrelated adults (18-65 years) (1,141 male, 1,188 female; 1,226 urban, 1,103 rural) were interviewed using a validated structured questionnaire. Ethics approval and informed consent from participants were obtained.

## Results

Headache was reported by 1,488 persons (crude annual 1-year prevalence 63.9%) with a mean age of 37±12 years, 58% females and 53% rural-dwelling. Only 24.7% (32.4% rural, 16.2% urban) had sought medical help. Of these, 80.6% had seen a primary-care doctor and 15.8% a specialist. Greater proportions of urban dwellers (38.6%) and females (17.7%) had consulted specialists. Consultation rates were higher for migraine (41.9% overall) but, even among those with high disability assessed as lost productive time by HALT questionnaire, did not exceed 50% (HALT grade 1: 27.3%; grade 2: 39.0%; grade 3: 45.7%; grade 4: 45.5%). Consultation rates were much higher for any headache occurring on ≥15 days/month (72.5%) and medication-overuse headache (78.6%).

## Conclusion

Despite the high prevalence of primary headache disorders, health-care utilisation is poor. The primary care physician is consulted most often, which is where headache services should be built. Structured headache services require primary-care physicians trained in managing headache disorders, facilitated links to secondary care when needed, but also improved awareness among people with headache so that they use them.

No conflict of interest.

